# Gender differences in psychological help-seeking attitudes: a case in Türkiye

**DOI:** 10.3389/fpsyg.2024.1289435

**Published:** 2024-03-07

**Authors:** Esra Güney, Ahmet Fatih Aydemir, Neslihan Iyit, Ömer Alkan

**Affiliations:** ^1^Department of Econometrics, Faculty of Political Sciences, Sakarya University, Sakarya, Türkiye; ^2^Department of International Trade and Logistics, Faculty of Economics and Administrative Sciences, Ataturk University, Erzurum, Türkiye; ^3^Department of Statistics, Faculty of Science, Selçuk University, Konya, Türkiye; ^4^Department of Econometrics, Faculty of Economics and Administrative Sciences, Ataturk University, Erzurum, Türkiye; ^5^Master Araştırma Eğitim ve Danışmanlık Hizmetleri Ltd. Şti., Erzurum, Türkiye

**Keywords:** psychological help-seeking, gender differences, public health, binary logistic regression, Türkiye

## Abstract

**Background/aim:**

Mental disorders pose a substantial public health challenge within the overall disease burden. This study aims to determine the factors associated with seeking psychological help among individuals experiencing depression according to gender differences in Türkiye.

**Methods:**

The study utilized microdata from Türkiye Health Survey conducted by the Turkish Statistical Institute in 2016, 2019, and 2022. Binary logistic regression analysis was employed to determine the factors associated with seeking psychological help.

**Results:**

The study’s findings reveal that variables such as survey year, age, education level, employment status, general health status, disease status, depression status, day service status in the hospital, daily activity status, tobacco use status, and alcohol use status are associated with the status of receiving psychological help.

**Conclusion:**

Gender-specific analysis indicated variations in the significance and impact of these variables among individuals seeking psychological help. In the development of preventive strategies for mental health protection, special attention should be given to factors associated with the psychological help-seeking behavior of both women and men. Prioritizing and addressing these factors will contribute to more effective mental health interventions.

## 1 Introduction

What life will bring remains an enigma and an unknown for people. The uncertainty of tomorrow can lead individuals to undergo troubled periods. People’s responses to difficulties in these trying times may vary. Some attempt to navigate these troubled periods with the support of family, friends, or close circles, avoiding professional help, while others choose to face these challenges (Nagai, 2022). Indeed, certain individuals find it challenging to cope with these problematic periods independently and opt to seek professional help (Nagai, 2015).

According to the Social Ecological Model, help-seeking behaviors are influenced by various factors, encompassing individual, interpersonal, community, and social aspects (Novak et al., 2023). The term “help-seeking,” defined as the act of seeking a solution to meet a person’s needs, has multiple interpretations (Cornally and McCarthy, 2011). In times of distress, help-seeking occurs when individuals in distress reach out to others for advice, information, treatment, or general support (Rickwood and Thomas, 2012). Generally, help-seeking is the process by which a person seeks resources to address self-defined needs, such as experiences of violence, health problems, and financial resources. The main components of help-seeking are categorized into three primary groups: the help-seekers, the individual(s) or group(s) with whom the help-seeker communicates (social network), and the outcomes of help-seeking, such as obtaining resources (Tull and Salusky, 2022).

Seeking professional help involves consulting with experts in the field when an individual is experiencing distressed moods, suicidal thoughts or behaviors, or undergoing mental distress processes that they cannot manage (Vayro et al., 2023). The ability of individuals to self-help and overcome psychological problems or distress is often limited. Overcoming problematic processes alone is generally not feasible. Seeking assistance not only from oneself but also from relatives and friends, who may be the first point of contact for solutions, may not be sufficient. Therefore, professional help becomes necessary in many cases (Pan and Hao, 2023) and is considered an expected behavior (Chan and Hayashi, 2010). Professional help-seeking is typically provided by health professionals such as general practitioners/family doctors, psychologists, psychiatrists/mental health specialists, and nurses (Vayro et al., 2023).

Mental including high morbidity, depression, psychoses, anxiety, and other related conditions, contribute to approximately 14% of the global disease burden (Seyfi et al., 2013). The initial step in treating mental disorders is the individual’s willingness to seek treatment. However, many people are reluctant to seek professional help for various reasons (Vogel et al., 2006; Rickwood and Thomas, 2012). Consequently, this percentage is predicted to be higher, as a significant number of individuals with mental disorders choose not to pursue professional assistance. Research findings indicate that this rate is indeed much higher. In a study conducted among adolescents in Türkiye, it was discovered that despite experiencing psychological difficulties, half of the participants, the majority, sought support from friends and family rather than seeking psychological help from specialists (Bilican, 2013).

The reluctance to seek psychological help when needed is not confined to any specific country (Pan and Hao, 2023). It has been noted that this situation is independent of the developmental status of countries. Even in nations with well-established access to health services, there is a significant reluctance among individuals with psychological problems to seek help from specialists (Rickwood and Thomas, 2012; Mojaverian et al., 2013).

People raised and moulded by their society learn specific social rules early on and pass them on to future generations. Cultural and gender norms play a significant role in teaching us about the appropriateness of discussing certain matters and shaping our expectations regarding different types of support. These teachings also affect our decision about seeking help and who we prefer to seek help from in the face of potential future problems (Vogel et al., 2006).

Fischer and Turner (1970) explained that individuals’ decisions to seek professional help are grounded in four factors: recognizing the need for psychological help, tolerance for stigmatization, interpersonal openness, and trust in mental health professionals (Fischer and Turner, 1970). In some studies within the field of psychotherapy, it has been suggested that men face specific challenges in acknowledging the need for professional help and actively seeking it. The study cited risks such as feelings of failure, loss of control, and perceptions of weakness as potential barriers for men in accepting and seeking professional assistance (Chan and Hayashi, 2010).

Professional help can serve as the most effective support for various mental health risks (Hedge et al., 2017). However, rejecting or delaying psychological help may lead to adverse consequences, including school failure or dropout, alcohol abuse, smoking, self-harm, suicide, or acute infections (Hao and Ieee, 2010). Consequently, numerous studies have sought to explore the reasons behind seeking or avoiding help and to identify the factors that either facilitate or hinder help-seeking when faced with social-psychological problems (Nagai, 2015). Studies have revealed that individuals’ attitudes and behaviors related to help-seeking are influenced by various factors, such as gender (Nagai, 2022; Shawcroft and Coyne, 2022), ethnic identity (Broman, 1987), profession (Burns and Buchanan, 2020), age, education, and marital status (Mackenzie et al., 2006).

In particular, the question of whether gender is an essential factor in seeking professional help has been debated in the past (Marrs et al., 2012; Molla, 2022). However, no consensus exists on which gender is more willing to seek help. Some studies have suggested that women tend to seek help less than men because they exhibit shy behaviors when seeking help for psychological difficulties (Molla, 2022). In contrast, other studies have indicated that women tend to seek psychological help more than men (Nam et al., 2010). Another finding suggests that women’s tendency to ask for help increases as their job rank rises, compared to men (Molla, 2022). Generally, it is thought that men are less willing to seek psychological help than women, possibly due to factors such as dominant male understanding and the media’s portrayal of men as strong (Shawcroft and Coyne, 2022).

Moreover, numerous studies have attempted to examine the reasons for help-seeking behavior in detail by focusing on a single gender group (Hammer et al., 2013; Shea et al., 2017; Parnell and Hammer, 2018). A study conducted on university students stated that the restrictive emotional characteristics of male students caused hesitation in seeking help. Additionally, it was noted that female students adopt more moderate attitudes towards help-seeking due to their openness to new ideas (Nam et al., 2010).

Various psychological disorders have been affecting societies for years, significantly impacting a considerable part of the population (Bayrakçeken et al., 2023). For various reasons, society often delays the identification of psychological illnesses and the implementation of appropriate treatment methods when seeking psychological help. While this delay may have various negative consequences, early diagnosis and intervention can help reduce the financial and moral costs associated with various psychological illnesses (Weber et al., 2011). Similarly, various psychological treatment methods have proven effective in addressing behavioral problems that arise at an early age. However, their effectiveness in older children and adolescents has been significantly diminished. Therefore, seeking psychological help should not be postponed, and its importance should be emphasized (Frick, 2012; Masroor et al., 2019).

Substance abuse and addiction, classified as psychological disorders that have been affecting societies for years, can serve as examples. Substance use and addictions, which impact a significant portion of society, can help alleviate the financial and moral costs that may arise through early diagnosis and intervention (Dermody et al., 2020).

In the literature, it is noted that gender plays a significant role in the inclination to seek psychological help. Many studies indicate that women tend to display more moderate behaviors when seeking professional help compared to men. However, this trend is observed not only in seeking psychological help but also in accessing general health services, where women make more requests than men (Saunders and Bowersox, 2007).

This study aims to investigate gender differences in seeking psychological help among individuals experiencing depression in Türkiye. In this context, the study attempts to examine the significance of gender in seeking professional help. The objective is to provide more precise information on whether women or men are more likely to seek professional help and whether gender is a crucial factor in seeking psychological help. Furthermore, the study underscores the importance of directing researchers, practitioners, health experts, and policymakers to explore the reasons for gender inequality in help-seeking behavior for challenges individuals may struggle to cope with.

It is believed that identifying the variables that prompt depressed Turkish citizens to seek psychological assistance will help concentrate psychological support services in areas where they are needed most, thereby enabling individuals to lead more comfortable lives. There have not been many studies conducted in this area in Türkiye. To address the question, “What factors affect individuals with depression in Türkiye receiving psychological help according to gender differences?” this study utilized a rich dataset to model the factors affecting individuals’ decisions to seek psychological help services in the country.

## 2 Methods

### 2.1 Data source

This study utilized the micro dataset of the Türkiye Health Survey (THS) conducted by the Turkish Statistical Institute (TurkStat) in 2016, 2019, and 2022. Official permission was obtained from the Turkish Statistical Institute to use the micro dataset from THS. Additionally, a “Letter of Undertaking” was provided to the Turkish Statistical Institute for the use of the data pertinent to this study.

The THS, conducted by TurkStat and encompassing responses from individuals residing in various regions of Türkiye, accurately reflects the social and demographic structure of the country (Alkan et al., 2023b). The THS was initiated to provide health indicators not captured by the administrative registration system, serving as a data source for decision-makers and researchers. It aims to gather information on health indicators, which constitute a significant portion of the developmental indicators reflecting a country’s level of development. This survey is crucial as the first study that comprehensively portrays the country and fulfils both international and national needs, enabling comparisons in terms of a study that sheds light on (TurkStat, 2016, 2020).

The THS was initially conducted by TurkStat in 2008, and subsequently, it was carried out periodically every 2 years until 2016. The THS provides numerous indicators on health, covering the health conditions of infants, children, and adults, utilization of health services, difficulties faced during daily activities, and habits related to cigarette and alcohol use for individuals aged 15 and over. The survey encompassed all individuals residing in Türkiye, excluding the institutional population (such as soldiers, individuals in dormitories, prisons, long-term care hospitals, homes for the elderly, etc.) and small settlements where a sufficient number of sample households cannot be reached (small villages, hamlets, etc.) (TurkStat, 2016, 2020).

A stratified two-step cluster sampling method is employed. The criterion for external stratification is the rural–urban division, where settlements with a population of 20,000 or less are considered rural, and those with a population of 20,001 or above are considered urban. The first-stage sampling unit comprises blocks randomly chosen with selection proportional to the size of the clusters (blocks), each containing an average of 100 addresses. The second-stage sampling unit involves systematically selecting household addresses from each chosen cluster (TurkStat, 2016, 2020).

**Figure 1:**
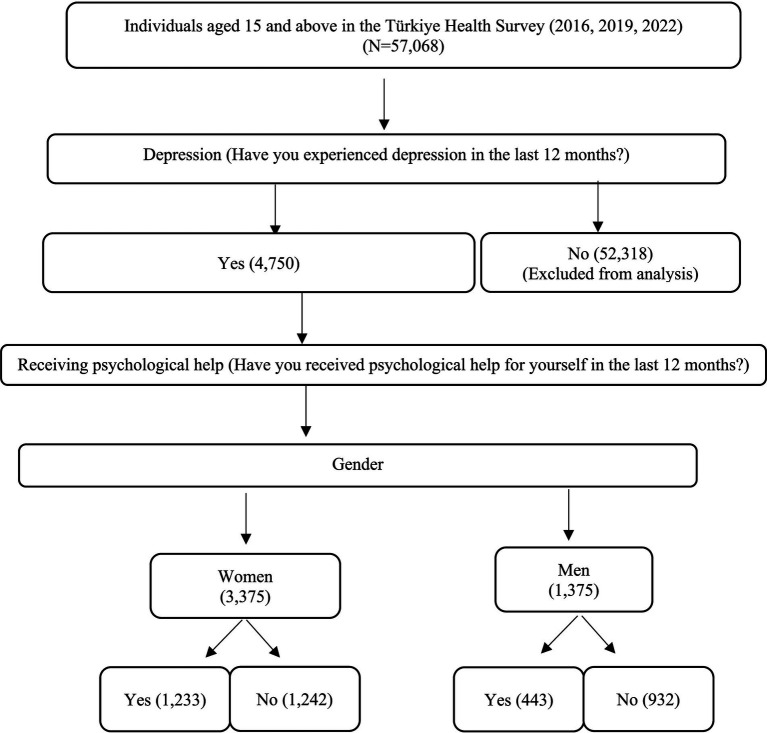
Sample selection process to determine effective factors in psychological help and determining the dependent variable.

The dataset used in this study is derived from the Health Survey conducted by the TurkStat in 2016, 2019, and 2022. The most recent available THS data shared by TurkStat is from 2022. The selection process of the sample included in the study is outlined in Figure 1.

### 2.2 Outcome variables

In the THS, individuals who participated in the survey were asked the following questions regarding their psychological help in the last 12 months as of the survey period: “Have you seen a psychologist for yourself in the last 12 months?,” “Have you seen a psychotherapist for yourself in the last 12 months?,” and “Have you seen a psychiatrist for yourself in the last 12 months?”

Individuals participating in the study received psychological help if they experienced one or more of the situations mentioned above, and they did not receive psychological help if they did not experience any of them. As a result, the dependent variable in the study is the status of individuals receiving psychological help, coded 1 if they received psychological help and 0 if they did not.

### 2.3 Independent variables

The independent variables included in the study comprise the variables available in the THS and those identified through literature research. Demographic and socioeconomic variables related to the participants include gender (female, male), age (15–24, 25–34, 35–44, 45–54, 55–64, and 65+), education level (illiterate, primary school, elementary school, high school, university), employment status (working, not working). Other factors that may be associated with receiving psychological help include year (2016, 2019, 2022), general health status (very good/good, moderate, bad/very bad), being sick (yes, no), experiencing depression (yes, no), receiving day hospital services (yes, no), daily activity status (mostly sitting or standing, mostly walking or work requiring moderate physical strength, mostly heavy work or work requiring physical strength), tobacco use status (yes, no), and alcohol use status (yes, no).

All the independent variables used in the present study are categorical. Ordinal and nominal variables were defined as dummy variables to observe the effects of categories for all variables included in the binary logistic regression (Demir et al., 2022; Alkan et al., 2023c; Ünver et al., 2023a).

### 2.4 Analysis method

Firstly, the participants’ status of receiving psychological help and the frequencies and percentages of the independent variables were obtained. Binary logistic regression analysis was chosen as the model for this study. The reason for selecting this analysis is that the dependent variable has a categorical structure (Baskan and Alkan, 2023; Ünver and Alkan, 2023; Kılıçarslan et al., 2024). The dependent variable in the model has two categories, measuring the status of receiving psychological help. Therefore, the model was estimated using binary logistic regression analysis. In the study, binary logistic regression analysis was conducted to determine the risk factors that influence gender differences in receiving psychological help.

## 3 Results

### 3.1 Descriptive statistics

The findings regarding the factors associated with the status of receiving psychological help in Türkiye by gender are presented in Table 1. Women constitute 71.1% of the sample. Among the participants, 41.6% of women and 35.4% of men, the highest percentage in the education level category, are primary school graduates. The age range of 45–54 represents 22.1% of the individuals in the study, with rates of 22.5% for women and 21.1% for men. Regarding tobacco and alcohol use, 37.9% of participants reported tobacco use, and 14.6% reported alcohol use. These rates were 32.3 and 9.4% for women and 51.9 and 27.3% for men, respectively.

**Table 1:** Findings related to the factors associated with receiving psychological help according to gender.

Variables		Whole Model		Female		Male	
		*n*	%	*n*	%	*n*	%
Year	2016	1,412	29.7	1,021	30.3	391	28.4
	2019	1,701	35.8	1,224	36.3	477	34.7
	2022	1,637	34.5	1,130	33.5	507	36.9
Age	15–24	445	9.4	298	8.8	147	10.7
	25–34	63	13.3	415	12.3	215	15.6
	35–44	967	20.4	693	20.5	274	19.9
	45–54	1,048	22.1	758	22.5	290	21.1
	55–64	873	18.4	649	19.2	224	16.3
	65+	787	16.6	562	16.7	225	16.4
Education level	Illiterate	755	15.9	662	19.6	93	6.8
	Primary school	1,892	39.8	1,405	41.6	487	35.4
	Middle school	685	14.4	424	12.6	261	19
	High school	793	16.7	506	15	287	20.9
	University	625	13.2	378	11.2	247	18
Employment status	Not working	1,253	26.4	643	19.1	610	44.4
	Working	3,497	73.6	2,732	80.9	765	55.6
General health status	Very good/Good	1,231	25.9	816	24.2	415	30.2
	Moderate	2,292	48.3	1,686	50	606	44.1
	Bad/Very bad	1,227	25.8	873	25.9	354	25.7
Disease status	No	270	5.7	172	5.1	98	7.1
	Yes	4,480	94.3	3,203	94.9	1,277	92.9
Day service status at the hospital	No	1,240	26.1	851	25.2	389	28.3
	Yes	3,510	73.9	2,524	74.8	986	71.7
Daily activity status	Mostly sitting or standing	2,829	59.6	2,058	61	771	56.1
	Mostly walking or work requiring moderate physical exertion	1,767	37.2	1,261	37.4	506	36.8
	Mostly heavy labor or work requiring physical strength	154	3.2	56	1.7	98	7.1
Tobacco use status	No	2,948	62.1	2,286	67.7	662	48.1
	Yes	1,802	37.9	1,089	32.3	713	51.9
Alcohol use status	No	4,058	85.4	3,058	90.6	1,000	72.7
	Yes	692	14.6	317	9.4	375	27.3
Gender	Male	1,375	28.9	-	-	-	-
	Female	3,375	71.1	-	-	-	-

### 3.2 Model estimation

Specific conditions were examined while estimating the model, and one of these is the multicollinearity problem (Alkan and Güney, 2021; Ünver et al., 2023b). The multicollinearity problem is relevant for multivariate regression analyses and investigates whether there is a strong or complete connection between independent variables. In this context, multicollinearity among the independent variables in the study, analyzed with the binary logistic regression model, was assessed using the variance inflation factor (VIF). It is stated that if the VIF values of the variables are 5 and above, there is a moderate degree of multicollinearity between the variables, while values of 10 and above cause a high degree of multicollinearity. In this study, no variable caused multicollinearity among the independent variables (Alkan et al., 2023a; Alkan and Kavalcı, 2023; Güney et al., 2023).

The results of the estimated binary logistic regression model are presented in Table 2. In all the models estimated for all individuals participating in the study, it is observed that individuals are related to the status of receiving psychological help according to gender.

**Table 2:** Estimated model results of the factors related to the status of receiving psychological help according to gender.

Variables	Whole model		Female		Male	
	*β*	Std. Error	*β*	Std. Error	*β*	Std. Error
Constant	−1.287^a^	0.237	−0.965^a^	0.291	−1.573^a^	0.396
Year (reference: 2016)						
2019	0.160^c^	0.192	0.155	0.108	0.182	0.173
2022	−0.103	0.090	−0.079	0.106	−0.173	0.168
*Age (reference: 65+)*						
15–24	0.540^a^	0.161	0.401^b^	0.197	0.819^a^	0.290
25–34	0.554^a^	0.147	0.522^a^	0.177	0.609^b^	0.274
35–44	0.535^a^	0.130	0.400^a^	0.151	0.822^a^	0.261
45–54	0.513^a^	0.125	0.465^a^	0.146	0.621^b^	0.250
55–64	0.345^a^	0.126	0.281^b^	0.145	0.510^b^	0.251
*Education level (reference: university)*						
Illiterate	−0.605^a^	0.150	−0.785^a^	0.178	−0.415	0.333
Primary School	−0.238^c^	0.122	−0.388^b^	0.153	−0.088	0.207
Primary education	−0.231^c^	0.134	−0.347^b^	0.172	−0.123	0.217
High School	−0.108	0.126	−0.331^b^	0.161	0.191	0.207
*Operating status (reference: not working)*						
Working	−0.333^a^	0.094	−0.336^a^	0.119	−0.326^b^	0.155
*General health status (reference: poor/very poor)*						
Very good/Good	−0.171	0.110	−0.140	0.134	−0.251	0.195
Moderate	−0.103	0.090	−0.040	0.106	−0.275	0.170
*Disease status (reference: no)*						
Yes	0.253	0.169	0.353^c^	0.209	0.130	0.279
*Day service status in the hospital (reference: no)*						
Yes	0.377^a^	0.083	0.272^a^	0.098	0.633^a^	0.159
*Daily activity status (reference: mostly sitting or standing)*						
Mostly walking or work requiring moderate physical exertion	−0.139^c^	0.077	−0.043	0.090	−0.352^b^	0.149
Mostly heavy labor or work requiring physical strength	−0.197	0.212	−0.019	0.318	−0.382	0.282
*Tobacco use (reference: no)*						
Yes	0.148^c^	0.076	0.103	0.091	0.242^c^	0.140
*Alcohol use (reference: no)*						
Yes	0.088	0.105	−0.092	0.144	0.261^c^	0.154
*Gender (reference: male)*						
Female	0.175^b^	0.082	-	-	-	-

^a^*p* < 0.01; ^b^*p* < 0.05; ^c^*p* < 0.10.

According to Table 2, the variables of survey year, age, education level (except high school), employment status, general health status, disease status, depression status, day service status in the hospital, daily activity status, tobacco use status, and alcohol use status are significant in the model estimated for individuals receiving psychological help. In the model estimated for women’s psychological help-seeking status, the variables of survey year, age (25–34 and 35–44), educational level (except high school), general health status (except middle school), disease status, depression status, day service status in the hospital, tobacco use status, and alcohol use status are significant. The model estimated that men’s psychological help-seeking status, age (except 55–64), educational level (except primary school and high school), employment status, general health status, illness status, depression status, day service status in the hospital, tobacco use status, and alcohol use status variables are significant.

The marginal effects of the factors related to the status of receiving psychological help according to gender are provided in Table 3.

**Table 3:** Marginal effects of factors related to receiving psychological help according to gender.

Variables	Whole model		Female		Male	
	ME	Std. Error	ME	Std. Error	ME	Std. Error
Year (reference: 2016)						
2019	0.101^c^	0.058	0.097	0.068	0.119	0.114
2022	−0.068	0.060	−0.051	0.069	−0.120	0.115
*Age (reference: 65+)*						
15–24	0.367^a^	0.108	0.268^b^	0.129	0.577^a^	0.204
25–34	0.375^a^	0.100	0.341^a^	0.115	0.442^b^	0.201
35–44	0.363^a^	0.090	0.267^a^	0.102	0.578^a^	0.190
45–54	0.350^a^	0.087	0.307^a^	0.098	0.450^b^	0.186
55–64	0.241^a^	0.089	0.191^c^	0.099	0.375^b^	0.187
*Education level (reference: university)*						
Illiterate	−0.399^a^	0.099	−0.495^a^	0.110	−0.291	0.241
Primary School	−0.147^b^	0.074	−0.227^a^	0.086	−0.059	0.138
Primary Education	−0.143^c^	0.083	−0.201^b^	0.099	−0.082	0.146
High School	−0.065	0.076	−0.191^b^	0.092	0.122	0.133
*Operating status (reference: not working)*						
Working	−0.220^a^	0.064	−0.221^a^	0.081	−0.219^b^	0.105
*General health status (reference: poor/very poor)*						
Very good/Good	−0.110	0.071	−0.089	0.085	−0.164	0.127
Moderate	−0.065	0.057	−0.025	0.066	−0.181	0.110
*Disease status (reference: no)*						
Yes	0.169	0.117	0.236	0.147	0.088	0.193
*Day service status in the hospital (reference: no)*						
Yes	0.251^a^	0.057	0.177^a^	0.065	0.441^a^	0.116
*Daily activity status (reference: mostly sitting or standing)*						
Mostly walking or work requiring moderate physical exertion	−0.090^c^	0.050	−0.027	0.057	−0.237^b^	0.102
Mostly heavy labor or work requiring physical strength	−0.129	0.142	−0.012	0.202	−0.258	0.199
*Tobacco use (reference: no)*						
Yes	0.095^b^	0.049	0.065	0.057	0.163^c^	0.095
*Alcohol use (reference: no)*						
Yes	0.056	0.067	0.059	0.094	0.171^c^	0.099
*Gender (reference: male)*						
Female	0.114^b^	0.054	–	–	–	–

^a^*p* < 0.01; ^b^*p* < 0.05; ^c^*p* < 0.10.

Women aged 15–24, 25–34, 35–44, 45–54, and 55–64 are 26.8, 34.1, 26.7, 30.7, and 19.1% more likely, respectively, to receive psychological help than those aged 65+ (the reference group). Illiterate, primary school, middle school, and high school education graduates are 49.5, 22.7, 20.1, and 21.9% less likely, respectively, to seek psychological help than university graduates. A working woman is 22.1% less likely to seek psychological help than others. A woman who receives day service in a hospital is 17.7% more likely to receive psychological help than others.

Men aged 15–24, 25–34, 35–44, 45–54, and 55–64 are 57.7, 44.2, 57.8, 45, and 37.5% more likely, respectively, to receive psychological help than those aged 65+ (the reference group). A working man is 21.9% less likely to seek psychological help than others. A man who receives daily services in a hospital is 44.1% more likely to receive psychological help than others. When the daily activity status of men is analyzed, a man who is in the group mostly walking or requiring moderate physical strength is 23.7% less likely to seek psychological help than a man who is in the group mostly sitting or standing, respectively. A man who uses tobacco is 16.3% more likely to seek psychological help than others. A man who consumes alcohol is 17.1% more likely to seek psychological help than others.

## 4 Discussion

This study employed binary logistic regression analysis to investigate the factors affecting the psychological help-seeking status of individuals in Türkiye according to their gender. For women, the variables of year, age, educational level, general health status, disease status, depression status, receiving day services in the hospital, and tobacco and alcohol use status are related to the psychological help-seeking status of individuals. In men, the variables of age, education level, employment status, general health status, disease status, depression status, receiving daily services in hospital, daily activity status, and tobacco and alcohol use status are related to the status of individuals receiving psychological help.

Studies consistently demonstrate that gender significantly affects help-seeking behavior (Good et al., 1989; Gim et al., 1990; Garland and Zigler, 1994; Barney et al., 2006; Hamid et al., 2009; Hao and Ieee, 2010; Koydemir-Ozden and Erel, 2010; Nam et al., 2010; Seyfi et al., 2013; Fry et al., 2014; Perenc and Radochonski, 2016; Rayan and Jaradat, 2016; Roskar et al., 2017; Menberu et al., 2018; Do et al., 2019; Cebi and Demir, 2020; Chen et al., 2020; Ramzi et al., 2021; Topkaya, 2021; Dalum et al., 2022; Gearing et al., 2022; Tull and Salusky, 2022; Nurdiyanto et al., 2023). Factors at all levels, such as demographic characteristics and negative perceptions of mental health treatment (individual), lack of social support (interpersonal), logistical barriers (community), and stigma (societal), are associated with reduced help-seeking among at-risk individuals (Novak et al., 2023). In an examination of attitudes towards seeking psychological help among university students, the study observed that a majority of participants believed that individuals seeking psychological help could be perceived as sick or crazy individuals. Many male participants expressed fears of societal rejection or being stigmatized as mentally unstable when seeking psychological help (Koydemir et al., 2010).

While there is ongoing discussion about the impact of gender on seeking psychological help, no definite judgment has been reached. A study conducted in China concluded that women were more likely to seek non-professional help than men, while men were more likely to seek professional help than women (Cheung, 1984). Given these inconclusive results, the relationship between gender and attitudes towards seeking psychological help requires further research. Consequently, the study determined that the significance and impact of the variables on seeking psychological help differed based on the gender of the individuals. These findings align with existing literature (Koydemir et al., 2010).

According to the findings, individuals who participated in the study in 2019 were more likely to receive psychological help than those who participated in 2016. Regarding general happiness level data by gender, while the rate of very happy individuals was 7.6 in 2016, this rate decreased to 6.6 in 2019. The rates for categories of happy, moderately happy, unhappy, and very unhappy were 53.8, 45.7; 28.3, 34.6; 8.8, 9.9; 1.6, 3.1 in 2016 and 2019, respectively. Upon analyzing the data, it was determined that the unhappiness data increased more in 2019. This situation may be considered to have contributed to the increased number of psychological help-seekers in Türkiye in 2016 (TUIK, 2020). Additionally, the political problems and terrorist attempts in Türkiye in 2016 may have impacted public health and prompted people to seek psychological help.

Working individuals are less likely to receive psychological help than non-working individuals. The analysis determined that this situation is also valid for men. Supporting this finding, various studies have been conducted (Picco et al., 2016; Chen et al., 2020). Additionally, studies indicate that occupational groups differ in their help-seeking behavior (Dalum et al., 2022). However, there is no definitive consensus on this issue. A study focusing on women found that working women were more sensitive to mental health disorders and more inclined to seek mental health services than housewives (Zalat et al., 2019).

The study determined that individuals aged 64 and under received psychological help more frequently than those in other age groups. The analyses revealed that this situation was consistent for both men and women. This finding aligns with the existing literature (Leaf et al., 1987; Al-Krenawi et al., 2004; Biddle et al., 2004; Segal et al., 2005; Koydemir-Ozden and Erel, 2010; Seyfi et al., 2013; Roskar et al., 2017; Chen et al., 2020). The study is in line with other studies suggesting a lower likelihood of seeking professional help at an older age (Greenley et al., 1987; Picco et al., 2016; Tull and Salusky, 2022). The reasons for the low likelihood of older individuals receiving psychological help are attributed to factors such as the absence of appropriate mental health systems and costs.

It was also found that, while older participants were less likely to seek professional help than younger participants, the same individuals were open to psychological help under appropriate conditions, and age was not a limiting factor in seeking help (Robb et al., 2003). However, there is no consistent information on this issue in the literature. Some studies have reported that older individuals are more open to seeking help (Barney et al., 2006; Rayan and Jaradat, 2016; Topkaya, 2021; Gearing et al., 2022), while others have found that help-seeking rates vary by gender, increasing with age in women and decreasing with age in men (Mariu et al., 2012).

It is observed that individuals with lower educational attainment, such as illiterate, primary school, and primary education graduates, are less likely to seek psychological help compared to university graduates. The analyses revealed that this situation was consistent for both men and women. The finding aligns with the literature, indicating a correlation between education levels and seeking professional help, with similar results found in previous studies (Barney et al., 2006; Perenc and Radochonski, 2016; Picco et al., 2016; Chen et al., 2020). Regardless of age, it was noticed that individuals with higher levels of education tended to have a more positive (Fischer and Cohen, 1972; Leaf et al., 1987).

While an unwell person is more likely to receive psychological help than others, this situation is also applicable to individuals receiving daily services in a hospital. Furthermore, individuals who rate their general health status as very good/good are less likely to seek psychological help compared to those with poor/very poor health status. The analysis revealed that this trend holds for both men and women. Individuals with chronic diseases may face limitations due to their conditions. Those who cannot perform regular activities in their daily lives due to chronic illnesses may experience psychological strain. It is also possible that such individuals are more inclined to seek psychological counselling services (Coskun et al., 2023).

Studies have revealed a relationship between symptoms of depression and the inclination to seek professional help (Garland and Zigler, 1994; Menberu et al., 2018). This situation is also true for openness to seeking professional help (Chen et al., 2020). Moreover, the severity of depressive symptoms has been reported to be associated with professional help-seeking behavior (Williams et al., 2008). Additionally, the negative relationship between depression and education indicates that the prevalence of depression tends to decrease as the level of education increases (Almeida et al., 2004). In a study aimed at determining rates and patterns of help-seeking for depression among Australian doctors, 60% of the participating doctors reported that those experiencing severe depression had sought some form of professional help. While this percentage may seem substantial, the fact that even doctors within the healthcare sector have reservations about seeking professional help for depressive episodes (attributed to concerns about confidentiality and potential impact on their careers) further underscores the significance of this issue (Ramzi et al., 2021).

Individuals whose daily activities mainly involve walking or work requiring moderate physical strength, and those predominantly engaged in heavy labor or work requiring physical strength, are less likely to seek psychological help than others. This situation is also valid for men. When past studies are analyzed, it is noted that activities such as yoga, meditation, or exercise have a positive impact on married working women, contributing to stronger mental health in those who engage in these activities (Panigrahi et al., 2014).

An individual who uses tobacco and alcohol is more likely to seek psychological help than others, as indicated by the analyses, and this holds true for both men and women. The finding aligns with existing literature suggesting that alcohol or tobacco use is associated with seeking professional help, with similar results found in previous studies. Previous studies concluded that there was a significant relationship between alcohol use and seeking professional help (Menberu et al., 2018). Furthermore, it is noteworthy that many individuals who are hesitant to seek help due to scepticism about professional assistance often suffer from alcohol disorders (Wells et al., 1994).

## 5 Conclusion

There may be various constraints on receiving psychological help, and people may resort to informal ways instead of professionals to cope with their existing problems. These constraints may include the intention to seek help, the fear of being stigmatized, and some demographic characteristics.

In the study conducted on students, people minimize their problems or do not seek professional help until they see their problems as severe (Koydemir et al., 2010). In addition to this situation, it is stated that people are distant from seeking help because they are afraid of being stigmatized (Menberu et al., 2018). In other words, the lower the fear of being stigmatized for seeking help, the higher the intention to seek professional help (Dagani et al., 2023). Even if this situation is usually evident in people with low education levels, it profoundly affects many segments of society (Roskar et al., 2017). The fact that even physicians working in the health sector, who know that psychological disorders are as severe as an ordinary physical health problem, continue to be concerned about the impact on confidentiality and career during the depression process has revealed how serious the situation is (Ramzi et al., 2021).

Especially in patriarchal societies, receiving psychological help can have different meanings. Considering that Türkiye has a patriarchal social structure, it is essential to investigate gender differences in receiving psychological help in Türkiye. Many studies have found that women are more likely to receive psychological help than men. Although there are various reasons for this, it can be shown that men are more closed to psychological problems and see it as a behavior that damages self-esteem (Roskar et al., 2017). In general, it is observed that men are less likely to seek help for health problems than women (Hernandez et al., 2014). This situation is also the case for receiving psychological help (Leong and Zachar, 1999). Although there are various reasons for this situation, the perception that men should be strong or the idea that mental illness is not as critical as physical illness can be listed (Ogueji and Okoloba, 2022).

In this study, several variables were significantly related to the psychological help-seeking behavior of individuals living in Türkiye. Consistent with many previous studies, women reported more positive attitudes towards seeking psychological help than men. Age ranges were also significantly associated with help-seeking behaviors for the whole model. Age ranges were also significantly associated with help-seeking behaviors for the whole model. Educational level was also a predictor of help-seeking behaviors, suggesting that university graduates (excluding high school) were more likely to engage in psychological help-seeking behaviors than individuals with other levels of education. In addition, general health, illness, depression, and hospital-day treatment were also significantly associated with help-seeking behaviors. In particular, it shows that alcohol and tobacco use increase the likelihood of individuals seeking psychological help.

To enhance public health and improve general health services, health courses can be offered to all segments of society, beginning in primary school. These courses aim to raise children’s awareness about negative situations that may lead to addiction, such as alcohol, tobacco, or drugs in the future. This proactive approach can help reduce the material and moral costs associated with these or similar addictions in the subsequent years. For older individuals, preventive measures can be implemented by investigating their environment and identifying potential addictive contacts, thus averting new cases of addiction. This strategy aims to keep the younger generation away from detrimental habits like tobacco, alcohol, and substances, fostering the development of healthy individuals.

In order to protect and improve the mental health of society, it is crucial to dispel the misconception that psychological disorders are fundamentally different from physical disorders. Emphasizing that those seeking psychological help are not labeled as “crazy” will help eliminate the associated prejudice. This shift in perception is expected to encourage individuals to seek psychological help without hesitation from an early age, facilitating access to early intervention and preventing the development of preventable psychological illnesses. Additionally, studying the health systems of developed countries can provide insights to develop alternative approaches for protecting and enhancing the mental health resources within the society.

## Data availability statement

The data analyzed in this study is subject to the following licenses/restrictions: the datasets presented in this article are not readily available because the data underlying this study is subject to third-party restrictions by the Turkish Statistical Institute. Data are available from the Turkish Statistical Institute (bilgi@tuik.gov.tr) for researchers who meet the criteria for access to confidential data. The authors of the study did not receive any special privileges in accessing the data. Requests to access the datasets should be directed to bilgi@tuik.gov.tr.

## Ethics statement

Ethical review and approval was not required for the study on human participants in accordance with the local legislation and institutional requirements. Written informed consent from the patients/participants or patients/participants’ legal guardian/next of kin was not required to participate in this study in accordance with the national legislation and the institutional requirements.

## Author contributions

EG: Writing – review & editing, Writing – original draft, Validation, Methodology, Investigation. AA: Writing – review & editing, Software, Data curation. NI: Formal analysis, Methodology, Validation, Writing – review & editing. ÖA: Writing – review & editing, Methodology, Formal analysis, Conceptualization.
